# A Modification of the Ostergren Model for Thermomechanical Fatigue Life Prediction of Die-Casting Die Steel

**DOI:** 10.3390/ma17235744

**Published:** 2024-11-24

**Authors:** Pengpeng Zuo, Xijuan He, Jie Ji, Xiaochun Wu

**Affiliations:** 1 School of Materials Science and Engineering, Jiangsu University of Science and Technology, Zhenjiang 212003, China; 2 Zhejiang Qingshan Iron and Steel Co., Ltd., Lishui 323903, China; 3 School of Materials Science and Physics, University of Mining and Technology, Xuzhou 221116, China; 4 State Key Laboratory of Advanced Special Steel, Shanghai University, Shanghai 200444, China; xijuanhe@163.com (X.H.); jijie9007@163.com (J.J.); xcwu@staff.shu.edu.cn (X.W.)

**Keywords:** thermomechanical fatigue (TMF), life prediction, die-casting die steel, Ostergren model

## Abstract

The Ostergren model is simple in form and widely used in engineering practice, also serving as the modeling basis of both the damage differentiation and crack propagation models. However, the shortcomings of the Ostergren model are that the modeling process is affected by thermomechanical fatigue (TMF) test parameters. To establish a TMF life normalized model, a modified Ostergren model based on hysteresis energy damage and TMF data for H13 steel was proposed. The model was successfully applied to TMF life prediction for 4Cr5Mo2V steel. The band of predicted life and test life is basically within the factor of 1.5. In summary, the modified Ostergren model is suitable for the TMF life prediction of Cr-Mo-V-type die-casting die steel.

## 1. Introduction

Thermomechanical fatigue (TMF) damage limits the life of die-casting dies because of oxidation–fatigue–creep complicated interactions [1,2]. According to incomplete statistics, thermal cracking due to TMF contributes to approximately 70% of die-casting die failures [3]. Therefore, improving TMF resistance is crucial for extending the service life of hot work dies. In recent years, research on the TMF behavior of hot work die steel has been steadily growing [1,2,3,4,5]. As conducting TMF tests is difficult, time-consuming, and costly, predicting fatigue life under varying temperatures is commonly derived from isothermal fatigue (IF) tests. However, the obvious difference between TMF and IF is the phase relationship between mechanical strain and temperature, which leads to this method having a large prediction deviation and low credibility [6,7,8,9]. Therefore, a perfect life model for TMF life prediction that is generally accepted does not currently exist [1,10,11,12,13,14]. Because TMF behavior is more suitable for the actual service conditions of die-casting dies, a reliable TMF life prediction model will hold greater guiding value for engineering applications.

At present, phenomenological models based on energy are more suitable for TMF life prediction, such as the Ostergren model [15]. It is simple in form and widely used in engineering practice, serving as the basis of both the damage differentiation model and crack propagation model. However, the shortcomings of the Ostergren model are that the modeling process is affected by TMF test parameters. To develop a normalized model for the TMF life of die-casting die steel, it is essential to account for the influence of test parameters on TMF experiments. Zhang Z. F. et al. [16,17] introduced a hysteresis energy model as an alternative to using stress or strain amplitudes to study extremely low cycle fatigue. This approach considers hysteretic energy generated during cyclic deformation as a measure of the total plastic work associated with fatigue damage.

Therefore, in order to establish a TMF life normalized model, we proposed a modified Ostergren model based on the hysteresis energy damage theory proposed by Zhang Z. F. et al. [16,17] and TMF data of H13 steel in this paper.

## 2. Materials and Methods

The tested steel was produced using electric-arc furnace melting, electro-slag remelting, and multi-directional forging and refined through heat treatment. Specimens were extracted from blooms of H13 forged hot work die steel. The nominal chemical composition (wt.%) of H13 steel is as follows: 0.39C, 0.99Si, 0.35Mn, 5.32Cr, 1.42Mo, 1.00V, 0.010P, 0.002S, and Fe balance. Cylindrical solid specimens were prepared with a gage diameter of 6.0 mm and a length of 12.0 mm, as detailed in Ref. [18]. Prior to TMF tests, specimen gauges were mechanically polished to prevent premature crack initiation from surface imperfections. Before final machining, all specimens underwent austenitizing at 1030 °C for 0.5 h in a vacuum furnace, followed by oil quenching. They were then tempered twice at 590–600 °C for 2 h, with air cooling after each tempering. The specimens had a hardness of 46.0 ± 1.0 HRC.

TMF tests were performed following ASTM E2368-24 [19] using a closed-loop servo-hydraulic test system (Landmark 370.10, MTS^®^ Systems Corp., Eden Prairie, Minnesota, MN, USA) with a maximum load capacity of 100 kN and a computer controller (FlexTest 40, MTS^®^ Systems Corp., Eden Prairie, Minnesota, MN, USA). Induction heating was provided by a 10 kW intermediate-frequency solid-state transmitter (TruHeat HF 3010, Huttinger Elektronik GmbH, Düsseldorf, Germany) while cooling was achieved through compressed air. Induction coils were tailored to ensure temperature uniformity along the gauge length, maintaining a variation within ±5 °C. The specimen ends were water-cooled using hydraulic grips, and a K-type Chromel–Alumel thermocouple spot-welded to the specimen for measured temperature. The axial mechanical strain was controlled using a high-temperature ceramic extensometer. Specific experimental methods are detailed in Ref. [18].

**Figure 1:**
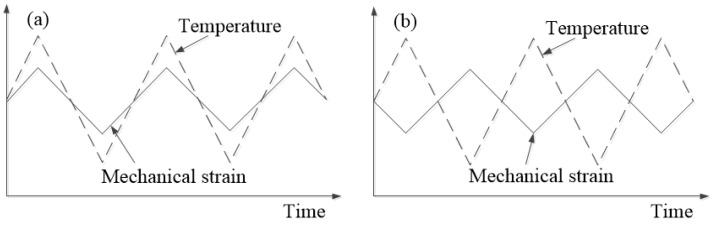
Schematics representation of TMF test waveforms [18]: (**a**) IP; (**b**) OP.

Phasing accuracy (*ϕ*) was assessed as the phase shift (in degrees) between the maximum temperature response on the specimen and the maximum mechanical strain response. A positive *ϕ* indicates the temperature maximum leads the strain maximum by 180° or less; otherwise, *ϕ* is negative. Two TMF test types were conducted to evaluate H13 steel’s behavior: in-phase (IP, *ϕ* = 0°) and out-of-phase (OP, *ϕ* = 180°) tests, both using triangular waveforms, as illustrated in Figure 1 [18]. All tests were strain-controlled, with a strain ratio ($R_{\varepsilon }$) of −1.

## 3. Results

Table 1 and Table 2 exhibit the TMF test results of H13 steel cycling at 400–700 °C and 200–600 °C, respectively. Table 3 shows the TMF test results of 4Cr5Mo2V hot work die steel cycling at 200–600 °C. All data in the three tables are taken from the stable hysteresis loop at the half-life cycle. These data will be adopted as the basis for the establishment of subsequent models.

Based on the Ostergren model, inelastic strain and cyclic tensile stress both play important roles in fatigue life [20]. The Ostergren model adopts net tensile hysteresis energy ($\sigma_{t}^{max}\cdot \Delta \varepsilon_{\mathrm{in}}$) to characterize fatigue damage. When time-dependent damage is excluded, the model equation is formulated as shown in Equation (1).

(1) $$\left(\sigma_{t}^{max}\cdot \Delta \varepsilon_{\mathrm{in}}\right)\cdot N_{f}^{m}=C$$

$\sigma_{t}^{max}$ is the maximum tensile stress, $\Delta \varepsilon_{\mathrm{in}}$ is the inelastic strain range, $N_{f}$ is the cycles at failure (i.e., fatigue life), and m and C are the material constants.

**Figure 2:**
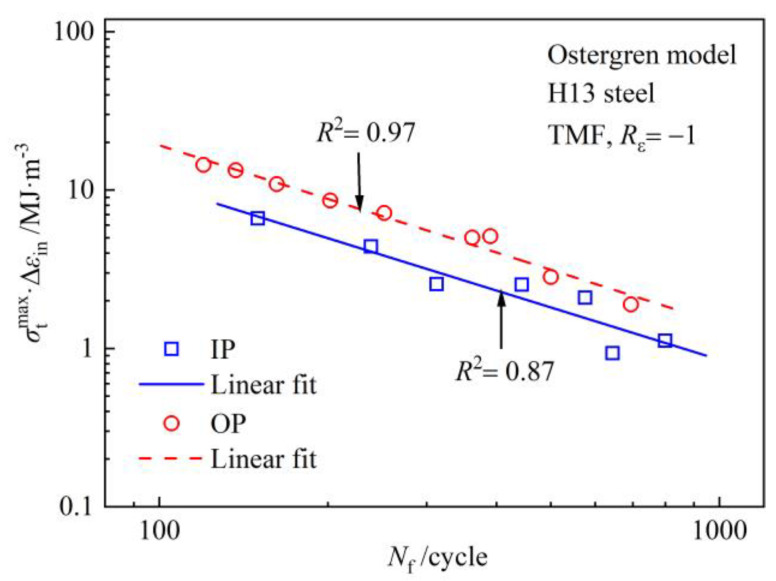
Fitting curves of the Ostergren model based on TMF data of H13 steel.

The test data of H13 steel are substituted into Equation (1), and double-logarithmic coordinate linear regression analysis of $(\Delta \sigma \cdot \Delta \varepsilon_{\mathrm{in}})-N_{f}$ is as shown in Figure 2. The correlation coefficients of the fitting curves indicate that this model reliably describes the TMF life of H13 steel. In particular, the description of the life behavior under OP-TMF simply fits the assumed curve better.

The m and C values of the IP-TMF and OP-TMF in Equation (1) can be obtained from the slope and intercept of the fitting curves in Figure 2. Then, the Ostergren models of H13 steel to predict life are established according to Equations (2) and (3).

(2) $$\mathrm{IP}-\mathrm{TMF}:\;\left(\sigma_{t}^{\max}\cdot \Delta \varepsilon_{\mathrm{in}}\right)\cdot {N_{f}}^{1.0984}=1672.55343$$

(3) $$\mathrm{OP}-\mathrm{TMF}:\;\left(\sigma_{t}^{\max}\cdot \Delta \varepsilon_{\mathrm{in}}\right)\cdot {N_{f}}^{1.12468}=3404.7090$$

As indicated above, if the original Ostergren model is adopted, the phase relationship between mechanical strain and temperature must be taken into account. As a result, different life prediction models will be established in different experimental parameters, which is troublesome and impractical.

To eliminate the influence of test parameters on TMF life prediction, a normalized TMF life model for die-casting die steel has been proposed. In the hysteresis energy model proposed by Zhang Z. F. et al. [16,17], a form of damage parameter (D) can be achieved from Equation (4).

(4) $$D_{i}=\frac{1}{N_{f}}={\left(\frac{W_{i}}{W_{0}}\right)}^{\beta }$$

Here, $D_{i}$ and $W_{i}$ represent the damage parameter and hysteresis energy for the  ith cycle, respectively, while $W_{0}$ denotes the intrinsic fatigue toughness. The parameter β is referred to as the damage transition exponent.

**Figure 3:**
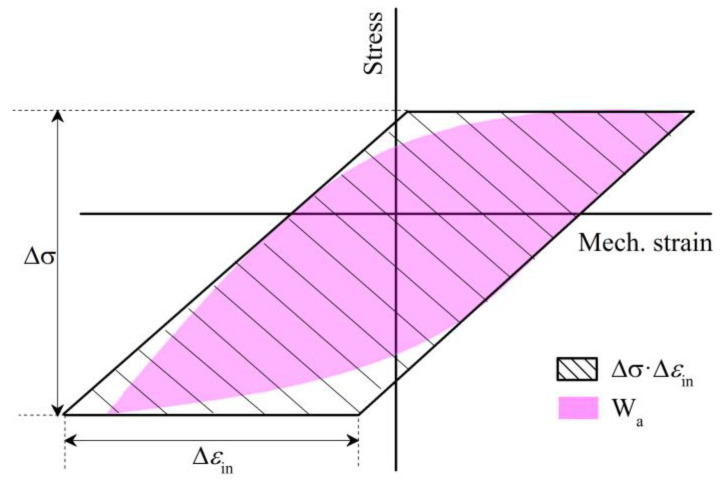
Definition of hysteresis energy and strain energy for low cycle fatigue.

Fatigue damage is influenced by both external and internal factors. External factors pertain to the loading conditions, such as stress and strain. Internal factors are associated with the material’s fatigue damage capacity and the cumulative energy ratio contributing to effective damage. Here, the above viewpoint is introduced into the TMF cycling damage. The plastic work ($W_{a}$) is used to describe the hysteresis energy shown in Figure 3. $W_{a}$ has a certain linear relationship with $\left(\Delta \varepsilon_{\mathrm{in}}\cdot \Delta \sigma \right)$ according to Equation (5).

(5) $$W_{a}=k\cdot (\Delta \varepsilon_{\mathrm{in}}\cdot \Delta \sigma )$$

Here, $\left(\Delta \varepsilon_{\mathrm{in}}\cdot \Delta \sigma \right)$ is the strain energy. k, referred to as the “shape factor”, represents the proportion of the hysteretic loop area in the parallelogram region.

If the damage parameter ($\sigma_{t}^{\max}\cdot \Delta \varepsilon_{\mathrm{in}}$) in the Ostergren model is changed to hysteresis energy (the hysteretic loop area), TMF damage at the half-life cycle can be described accurately and intuitively. Thus, the modified Ostergren model can be expressed as Equation (6).

(6) $$(k\cdot \Delta \sigma \cdot \Delta \varepsilon_{\mathrm{in}})\cdot {N_{f}}^{m}=C$$

Here, the shape factor (k) is about 0.84±0.0015, derived from a large quantity of experimental data based on Equation (5). The above equation can be rearranged into Equation (7).

(7) $$(0.84\cdot \Delta \sigma \cdot \Delta \varepsilon_{\mathrm{in}})\cdot {N_{f}}^{m}=C$$

Here, m and C are the material constants.

**Figure 4:**
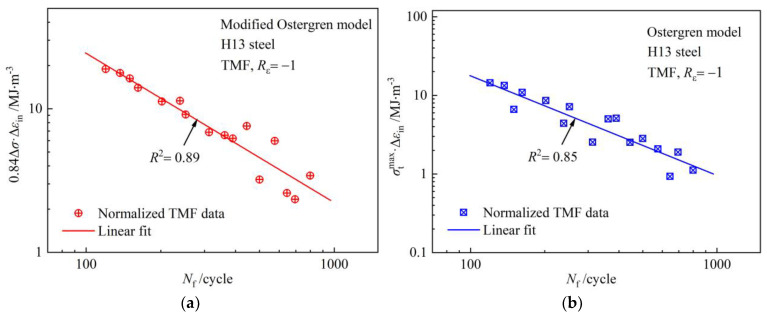
Fitting curves based on TMF data of H13 steel: (**a**) modified Ostergren model; (**b**) Ostergren model.

Regardless of how the TMF test parameters change, as long as the normalization concept is introduced, the TMF life can be predicted by the stress range and inelastic strain simply obtained from the stress–strain hysteresis loop. It simplifies the large number of physical parameters required in other models and achieves operability and practical value. Figure 4a presents double-logarithmic coordinate linear regression analysis of $(0.84\cdot \Delta \sigma \cdot \Delta \varepsilon_{\mathrm{in}})-N_{f}$ based on the TMF data of H13 steel. The material constants (m and C) are obtained from the slope and intercept of the fitting curve. When the values of m and C are substituted into Equation (7), the TMF life prediction normalization model (i. e., the modified Ostergren model) of die-casting die steel can be given as Equation (8).

(8) $$\left(0.84\cdot \Delta \sigma \cdot \Delta \varepsilon_{\mathrm{in}}\right)\cdot {N_{f}}^{1.03958}=2921.66219$$

For the purpose of comparing the modified Ostergren model and Ostergren model, the fitting curve is also obtained in Figure 4b, and the normalized Ostergren model is also established according to Equation (9).

(9) $$\left(\sigma_{t}^{\max}\cdot \Delta \varepsilon_{\mathrm{in}}\right)\cdot {N_{f}}^{1.2652}=5999.43013$$

It can be seen from the correlation coefficients of the fitting curves in Figure 4 that the data points of the modified Ostergren model have less dispersion and higher fitness.

**Figure 5:**
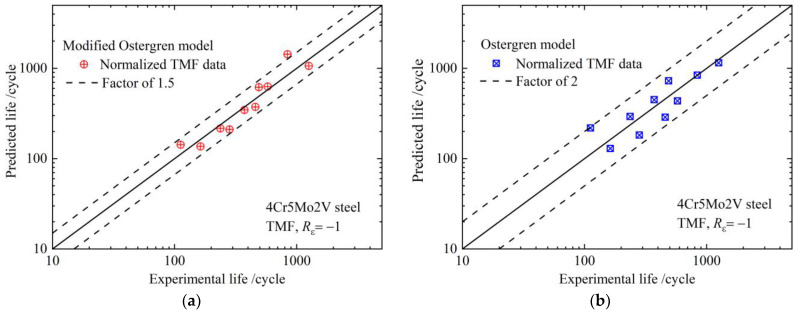
TMF life prediction reliability of normalized models of die-casting die steel: (**a**) modified Ostergren model; (**b**) Ostergren model.

To further validate the predictive capability of the two normalized models, the TMF life of 4Cr5Mo2V steel was predicted by Equations (8) and (9) based on the data in Table 3. The fitting curve is presented in Figure 5.

The prediction reliability of the two normalized models was evaluated using the scatter band. The data points calculated using the normalized modified Ostergren model are nearly within a factor of 1.5 of the scatter band, as shown in Figure 5a. However, the data points calculated by the normalized Ostergren model are almost within a factor of 2.0 of the scatter band, as shown in Figure 5b. Additionally, this model is closer to the midline, suggesting that the modified Ostergren model is more accurate for predicting the TMF life of die-casting die steel.

## 4. Conclusions

In conclusion, based on hysteretic energy damage and the TMF data of H13 hot work die steel, a modified Ostergren model for predicting the TMF life of die-casting die steel is proposed, as given in Equation (8).

The modified Ostergren model accurately predicts the TMF life of 4Cr5Mo2V hot work steel. In addition, the scatter band for predicted life and experimental life are within a factor of 1.5.

## Figures and Tables

**Table 1:** TMF test results of H13 steel cycling at 400–700 °C (half-life cycle).

Loading Type	$\left(\Delta \varepsilon_{m}/2\right)$/%	$N_{f}$	$\sigma_{t}^{max}$/MPa	Δσ/MPa	$\Delta \varepsilon_{\mathrm{in}}$/%
IP	0.5	800	171.9	626.9	0.65
	0.7	576	214.6	733.7	0.97
	0.9	444	211.9	758.0	1.19
OP	0.5	500	462.2	627.2	0.61
	0.7	390	526.4	762.2	0.97
	0.9	252	531.1	804.5	1.35
	1.1	162	663.3	1016.3	1.64
	1.3	120	720.5	1125.8	2.00

In Table 1, $\Delta \varepsilon_{m}/2$ is mechanical strain amplitude; Δσ is stress range.

**Table 2:** TMF test results of H13 steel cycling at 200–600 °C (half-life cycle).

Loading Type	$\left(\Delta \varepsilon_{m}/2\right)$/%	$N_{f}$	$\sigma_{t}^{max}$/MPa	Δσ/MPa	$\Delta \varepsilon_{\mathrm{in}}$/%
IP	0.5	645	423.8	1400.2	0.22
	0.7	313	478.7	1539.9	0.53
	0.9	239	523.2	1609.4	0.84
	1.1	150	606.3	1775.6	1.09
OP	0.5	695	948.4	1393.0	0.20
	0.7	362	982.1	1525.7	0.51
	0.9	202	1144.6	1784.7	0.75
	1.1	137	1128.7	1791.1	1.18

**Table 3:** TMF testing results of 4Cr5Mo2V steel cycling at 200–600 °C (half-life cycle).

Loading Type	$\left(\Delta \varepsilon_{m}/2\right)$/%	$N_{f}$	$\sigma_{t}^{max}$/MPa	Δσ/MPa	$\Delta \varepsilon_{\mathrm{in}}$/%
IP	0.5	1258	473.6	1454.9	0.17
	0.6	490	534.1	1611.5	0.27
	0.7	373	519.3	1560.4	0.51
	0.9	237	651.3	1852.8	0.70
	1.1	112	609.3	1851.5	1.08
OP	0.5	841	999.7	1511.4	0.12
	0.6	579	1099.9	1712.1	0.25
	0.7	459	1012.6	1592.0	0.46
	0.9	282	1151.2	1851.4	0.72
	1.1	163	1171.2	1919.9	1.09

## Data Availability

The original contributions presented in the study are included in the article; further inquiries can be directed to the corresponding authors.
